# Concentration-Dependent, Size-Independent Toxicity of Citrate Capped AuNPs in *Drosophila melanogaster*

**DOI:** 10.1371/journal.pone.0029980

**Published:** 2012-01-04

**Authors:** Giuseppe Vecchio, Antonio Galeone, Virgilio Brunetti, Gabriele Maiorano, Stefania Sabella, Roberto Cingolani, Pier Paolo Pompa

**Affiliations:** 1 Italian Institute of Technology, Center for Bio-Molecular , Arnesano (Lecce), Italy Nanotechnologies@UniLe; 2 Italian Institute of Technology, Central Research Laboratories, Genova, Italy; University of California, Merced, United States of America

## Abstract

The expected potential benefits promised by nanotechnology in various fields have led to a rapid increase of the presence of engineered nanomaterials in a high number of commercial goods. This is generating increasing questions about possible risks for human health and environment, due to the lack of an in-depth assessment of the physical/chemical factors responsible for their toxic effects. In this work, we evaluated the toxicity of monodisperse citrate-capped gold nanoparticles (AuNPs) of different sizes (5, 15, 40, and 80 nm) in the model organism *Drosophila melanogaster,* upon ingestion. To properly evaluate and distinguish the possible dose- and/or size-dependent toxicity of the AuNPs, we performed a thorough assessment of their biological effects, using two different dose-metrics. In the first approach, we kept constant the total surface area of the differently sized AuNPs (Total Exposed Surface area approach, TES), while, in the second approach, we used the same number concentration of the four different sizes of AuNPs (Total Number of Nanoparticles approach, TNN). We observed a significant AuNPs-induced toxicity *in vivo*, namely a strong reduction of *Drosophila* lifespan and fertility performance, presence of DNA fragmentation, as well as a significant modification in the expression levels of genes involved in stress responses, DNA damage recognition and apoptosis pathway. Interestingly, we found that, within the investigated experimental conditions, the toxic effects in the exposed organisms were directly related to the concentration of the AuNPs administered, irrespective of their size.

## Introduction

The rapid expansion of nanotechnology is producing a huge assortment of nanoparticles that differ in chemical composition, size, shape, surface charge and chemistry, coating and dispersion status [1]. Such nanostructured materials are rapidly entering in the production cycles of a wide range of commodities, including pharmaceutics, cosmetics and biomedical products, generating increasing questions about possible risks for human health and environment [2], [3]. Nanoparticles, however, exhibit peculiar physicochemical properties that may also represent major obstacles for the development of reliable and comparable protocols for correct nanotoxicity assessment. In this frame, it is now widely recognized that a detailed nanomaterials characterization is crucial to avoid the occurrence of dissimilar results in the evaluation of their toxicity, also due to their typical colloidal instability, propensity to aggregation, and large size dispersion. Similarly, the choice of the dose metrics is also of great importance, although contrasting results and hypotheses have been reported until now [4]–[6]. In addition, several studies have demonstrated the existence of biophysicochemical interactions at the nano–bio interface, such as protein corona formation, which may have a significant role in the intracellular uptake of nanomaterials, with possible influences on the toxicity outcomes [6]–[8]. All these issues generally make the comparison of the experimental results from different nanotoxicological studies rather difficult [9], [10]. In this context, it is important to define a rigorous strategy to study the complex interactions occurring between nanostructured materials and living systems, by a deep nanomaterial characterization followed by a well established *in vivo* experimental procedure. This approach may be useful to define a correct experimental route [11], [12] that may provide a deeper understanding in the definition of dose, dose metrics, and bio-kinetics in the case of NPs.

In this work we investigated the *in vivo* effects of metrologically controlled gold nanoparticles (AuNPs) of different size (5, 15, 40 and 80 nm, with a size dispersion ≤6%) on the model organism *Drosophila melanogaster,* upon ingestion. The investigation about nanoscale gold is of great interest because it is largely used in several bio-medical applications, including drug delivery [13]–[17], photothermal therapy [18], probe and cell imaging [19]–[21]. However, a large number of recent studies [22]–[29] is increasingly showing that AuNPs are significantly toxic [30]. We used *Drosophila* as model organism because it offers several advantages, such as short lifespan, high genetic and functional homology with higher organisms, and high efficiency for massive screening [31]. For these reasons, *Drosophila* was behind many of the fundamental advances in genetics, molecular and developmental biology in the last century [32]–[33]. More recently, it was also successfully used to reveal the biological activity of several chemicals encountered through environmental exposure [34]–[36], resulting the predominant alternative model to mammalian ones to study human diseases [37]–[44] and to assess the toxicity of chemical compounds and nanomaterials [34], [35], [45]. Notably, we have recently demonstrated the toxic effects of 15 nm citrate capped AuNPs both *in vitro* and in *Drosophila* upon ingestion [45], [46]. In the present work, we expanded our investigation by analyzing the role of the NPs size and concentration in determining possible adverse effects. In particular, the purpose of this study was twofold: *i)* to assess the *in vivo* toxic effects of differently sized AuNPs through a detailed analysis of several biological aspects (evaluation of lifespan, fertility, cellular stress by Reactive Oxygen Species formation, genotoxicity by TUNEL assay, and genes expression profiling by Real-Time qPCR to evaluate the response to stress stimuli, such as DNA damage checkpoints and apoptosis); *ii)* to understand the importance of the physical parameters that influence the toxicity of AuNPs in the 5÷80 nm range. To this aim, we compared the effects of the surface area and concentration of the AuNPs by using two experimental approaches in parallel: the “Total Exposed Surface area” approach (TES) and the “Total Number of Nanoparticles” approach (TNN). In the TNN approach, we used the same concentration number of the differently sized AuNPs, while in the TES approach we normalized the AuNPs concentration to have the same surface area for the different 5÷80 nm sizes administered to the flies.

## Materials and Methods

### AuNPs synthesis and characterization

All glassware and the magnetic stir-bar were washed thoroughly with aqua regia (HCl and HNO_3_ in a 3:1 volumetric ratio). Colloidal 5 nm citrate-capped AuNPs were prepared in a round bottom flask with 100 mL ice-cold aqueous solution containing 0.25 mM HAuCl_4_ (Sigma-Aldrich) and 0.25 mM trisodium citrate (Sigma-Aldrich). Then 0.6 mL of ice-cold freshly prepared 0.1 M NaBH_4_ (Sigma-Aldrich) solution was added while stirring. The solution turned red-brown immediately after the addition of the reducing agent, indicating particles formation. Here, citrate serves only as a capping agent since it cannot reduce the gold salt at this temperature (4°C). Colloidal 15 nm citrate-capped AuNPs were synthesized by the classical Turkevich–Frens method [47], [48], using sodium citrate as reducing agent. Briefly, 150 mL of 0.25 mM aqueous solution of HAuCl_4_ was heated to boil while stirring. Then, 2.8 mL of 1% aqueous solution of sodium citrate were added. The solution was kept gently boiling until a red wine color appeared. AuNPs of 40 and 80 nm were prepared according to a two-step seed-mediated method [49] which allows the enlargement of 15 nm AuNPs (seeds) for the property of NH_2_OH to efficiently reduce Au^3+^ to bulk metal in the presence of Au surface [50]. The synthesis was performed by adding 2 mL of aqueous 40 mM hydroxylamine sulfate (Sigma-Aldrich) and different numbers of 15 nm AuNPs (seeds) into 200 mL aqueous solution. The solution was kept under vigorous stirring and then 25 mL of 2 mM aqueous solution of HAuCl_4_ was dropwise added to seeds solution (1 mL/min). After the addition of HAuCl_4_ solution was finished, stirring was continued for 30 min and then 12 mL of 1% aqueous solution of trisodium citrate was injected to stabilize AuNPs by the weak capping effect of such chemical. To minimize the presence of solvent and unreacted reagents, all the solutions were immediately centrifuged for 15 min, then 5, 15, 40 and 80 nm AuNPs were suspended in ultrapure, sterile water. Before their use, NPs were filtered using a 0.22 µm syringe filters (Fluorophore PTFE membrane, purchased form Millipore Corp.) under a laminar flow biological safety cabinet, to ensure sterility.

To obtain essential information on AuNPs size and shape, TEM images were carried out. The 300 mesh carbon coated copper grid was casted with few drops of citrate-capped AuNPs and vacuum dried. TEM images of each sample were collected using a JEOL 1011 transmission electron microscope with an accelerating voltage of 100 kV. UV–Vis spectra were recorded using a Cary 300 Bio double-beam spectrophotometer at 300 nm/min scanning rate from 400 to 850 nm. The AuNPs concentrations were measured using the molar extinction coefficients measured at the wavelength of the plasmon peak [51], [52]. Further characterizations were performed by Dynamic Light Scattering (DLS) and Zeta potential analyses using a Zetasizer Nano-ZS instrument (Malvern Instruments) equipped with a 4.0 mV-He-Ne 633 nm laser.

### Drosophila melanogaster strain and culture conditions

The flies and larvae of wild-type *Drosophila melanogaster* (Oregon R+) were cultured at 24±1°C on standard *Drosophila* food, containing agar, corn meal, sugar, yeast and nepagin (methyl-p-hydroxybenzoate).

### AuNPs exposure

AuNPs were formulated in the *Drosophila* diet. Four different sizes (5, 15, 40 and 80 nm) of AuNPs were dispersed in the food and used for experiments as described previously [45]. Briefly, the solution containing AuNPs was added to the food before solidification, mixed strongly and finally poured into vials. With the same modality, we prepared food with the AuNPs supernatant (SN), obtained by centrifugation of the solutions of the differently sized AuNPs (mixed together after centrifugation). This preparation was used to exclude the presence of toxic compounds in the solution containing the AuNPs. Moreover, to evaluate the dispersion of AuNPs mixed in the *Drosophila* food, we carried out TEM analyses. The 300 mesh carbon coated copper grid was casted with few drops of food and then vacuum dried. The TEM images of each sample were collected using a JEOL 1011 transmission electron microscope, with an accelerating voltage of 100 kV, and showed that the AuNPs do not significantly aggregate (Figure S2).

For the TES approach we maintained constant the total surface area of all the sizes of AuNPs (4.25×10^10^ nm^2^/µL), while, for the TNN approach we maintained constant at 100 pM (6.02×10^7^ NPs/µL) the concentration of all the sizes of AuNPs. Relationships between TES and TNN for the two approaches are shown in Table S1. In these experiments the dose of gold ingested by Drosophila ranges from 0.114 to 467 µg/g (each *Drosophila* ingests, on average, a volume of 1.50±0.04 µL of food per day) [53].

### Lifespan experiments

For longevity analyses, newly eclosed flies were collected and housed at a density of 20 males and 20 females, separately, per each vial. At least 10 vials were used per treatment (total of 100 males and 100 female flies per lifespan) for a total number of 1,200 flies in TNN experiment and 1,200 in TES experiment. Flies were transferred into fresh food every 3–4 days, and dead flies were counted every day until all died. We carried out this experiment using normal food, treated food containing AuNPs supernatant (SN) and treated food containing AuNPs of different sizes.

### Fertility and reproductive performance

Fertility and reproductive analyses were performed as previously reported [45]. Briefly, virgin flies emerging from control, SN and AuNPs treated food (of TES or TNN approach) were isolated and pair mated in normal food vials. The total number of flies eclosed from the eggs laid during these ten days of pair mating was counted. The mean number of flies emerged per pair for ten days gave a measure of the reproductive performance.

### Measurement of ROS

Molecular oxygen is the key to aerobic life but it may also be converted into cytotoxic byproducts referred to as reactive oxygen species (ROS). In addition to their involvement in the normal metabolic activities, ROS have been reported to play a major role in the toxicity of several xenobiotics, including metals and pesticides [54].

To measure the intracellular ROS level in *Drosophila*, we used the non-fluorescent 2,7-dichlorofluoresceindiacetate (DCF-DA, Sigma-Aldrich), a cell permeable dye that can be converted into fluorescent 2,7-dichlorofluoroscein (DCF) by interacting with hydrogenperoxide [55]. Twenty five-day-old flies were homogenized in tubes containing 1 mL PBST (PBS containing 0.1% Tween-20). The homogenate of each sample was divided in two different vials. The first vial was transferred into a 96-well plate. After adding 50 µM DCF-DA to the samples, the plate was read every 5 min for 15 min with a fluorescent microplate reader (FLUOstar Optima, BMG Laboratory, Offenberg, Germany) for the quantification of fluorescence (485 nm excitation, 520 nm emission). The second vial was used for protein crude extract quantification. Following centrifugation at 2300 g for 15 min at 4°C in the presence of a protease inhibitor, the supernatant was quantified by the Bradford method [56]. The amount of proteins in the crude extraction was used to normalize the relative fluorescence measured by DCFH-DA in each samples. Three independent experiments with 20 flies in each experiment were performed.

### TUNEL assay

Third instar larvae midgut were dissected in Ringer's Buffer and fixed as previously described [45]. Briefly, midgut was processed by Click-iT TUNEL Alexa Fluor647 Imaging Assay (Invitrogen), containing TdT enzyme and a modified dUTP. Then, midgut was washed twice with 3% BSA (Bovine Serum Albumin) in PBS for 2 minutes each and incubated with Click-iT reaction cocktail for 30 min at room temperature, in the dark. Finally, the samples were incubated for 15 min at room temperature with 1X Hoechst 33342 solution. These samples were characterized by confocal microscopy (Leica TCS-SP5 AOBS). Semi-quantitative analyses of TUNEL-positive nuclei were carried out by examining different intestinal tissues dissected from flies of all the treatments (20 different microscopic fields each) from three independent experiments.

### Quantitative Real-Time PCR Expression Profiling

Third instar larvae extracts were prepared by homogenizing larvae in groups of 10 in cold solution of RNAlater (SIGMA). Total RNA was isolated from flies using Tri-reagent (Sigma); the amount of RNA in each sample was determined by Nanodrop, and RNA quality was analyzed using agarose gel electrophoresis (1.2%). First-strand cDNA was prepared from 3 µg of total RNA using Enhanced Avian Reverse Transcriptase (Sigma Aldrich) and oligo(dT)_18_ primers in 20 µL reaction volume, and 2.5 µg were digested with RNase (Sigma Aldrich). Real-time quantitative PCR was performed with an ABI 7500 thermal cycler (Applied Biosystem) following manufacturer's suggestions and using SYBR Green-based detection of PCR products. Melting curves were examined after amplification to exclude the presence of unspecific amplification targets. For each gene we used 10 ng of cDNA mixed with 10 µL of 10× Express SYBR Green qPCR SuperMix premixed with ROX (Invitrogen), 2 µL of 4 µM gene specific primers mix and 7 µL of DEPC-treated water. Reaction conditions for all genes were: initial denaturation at 95°C for 10 min followed by 40 cycles of 15 s at 95°C, 1 min at 60°C. This program was followed by a melting curve program (60–95°C with a heating rate of 0.1°C/s and continuous fluorescence measurements). Relative expression was calculated by Applied Biosystem Software through ΔΔCt method, using RpL32 ribosomal RNA expression as an internal control for each sample. The primers used in Real-Time qPCR analysis were designed by on-line Primer-BLAST software of NCBI (the list is reported in Table S2).

### Statistical analyses

GraphPad Prism 5 statistical analyses software was used in all statistical analyses performed in this work (GraphPad Prism version 5.00 for Windows, GraphPad Software, San Diego California USA). In particular, the survival distributions (lifespan curves) were assessed in terms of significance using the non-parametric Log-rank (Mantel-Cox) Test; the TUNEL assays were evaluated by *t*-test; the Reactive Oxygen Species (ROS) measurement, and the fertility tests were analyzed by One-way ANOVA and compared to the control by Bonferroni post test. RT-qPCR results were analyzed by Two-way ANOVA, and all gene expressions were compared to the control by Bonferroni post test.

## Results and Discussion

In this study we used two experimental approaches (TES and TNN) to evaluate the toxic effects of differently sized (5, 15, 40, and 80 nm) and monodispersed citrate-capped AuNPs (see Figure S1 for characterization details) in *Drosophila melanogaster* upon ingestion. Both approaches were performed using AuNPs dispersed in the flies food, using a wide dose range (from 0.11 to 467 µg/g per day) (all the AuNPs concentrations used in each treatment are reported in Table S1). The biological effects of the AuNPs on the organisms were evaluated in terms of lifespan, fertility, reactive oxygen species (ROS) levels, DNA damage, and modification of the expression level of genes involved in response to stress, DNA damage recognition and apoptosis.

### Viability and fertility tests

**Figure 1:**
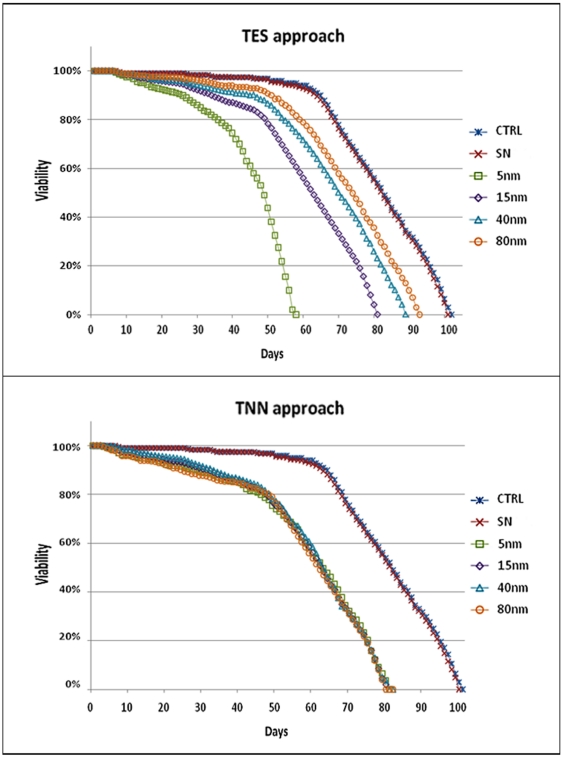
Lifespan curves of *Drosophila* flies nurtured with AuNPs treated food (5, 15, 40, and 80 nm) compared to two populations bred with normal food (CTRL) or supernatant treated food (SN). Fig. 1, top and bottom, are relative to TES and TNN approach, respectively. Experimental points represent the average from 5 independent experiments (the standard deviations are reported as the curve symbols size). The lifespan curves of both TES and TNN experiments were validated by the non-parametric log-rank (Mantel-Cox) test (see Table S3).

As a first step, we investigated the effects caused by AuNPs on *Drosophila* viability, performing lifespan studies relative to both approaches. The lifespan curves obtained from TES and TNN experiments are reported in Fig. 1. Experimental data highlight an unequivocally negative effect of AuNPs ingestion on *Drosophila* lifespan, revealing a significant toxicity of such nanomaterials (consistent with our recent observations on 15 nm AuNPs [45]). In particular, by analyzing the half-life (τ_50_) of the flies, it is possible to understand the contribution of the two physical parameters under study (i.e., concentration and size). Examining the TES experiments (Fig. 1, top), a different decrease of the viability of *Drosophila* can be clearly observed among the differently sized NPs. This indicates that the toxic effects are not directly related to the surface area of the AuNPs. In particular, the graph shows a higher toxic effect in the case of the smallest AuNPs, (5 nm, τ_50_ = 48 days) followed by 15, 40 and 80 nm AuNPs (τ_50_ = 62, 70, and 74 days, respectively). However, such apparent size-dependent toxicity is due to the fact that, in the TES approach, the AuNPs concentration increases with decreasing their size. In particular, in these experiments the concentration of 5 nm AuNPs is more than two orders of magnitude higher than that of 80 nm AuNPs (900 *vs.* 3.5 pM for 5 and 80 nm AuNPs, respectively, see also Table S1). This finding is further confirmed by the TNN experiments, in which the concentration of the AuNPs is kept constant (100 pM) for all the AuNPs sizes. In this test (Fig. 1, bottom), in fact, the lifespan decrease was the same for all the NPs sizes (τ_50_ = 62 days). This means that the *Drosophil*a viability is directly affected by the number of AuNPs formulated in the food, regardless of their size/surface area (in the 5-80 nm size range).

**Figure 2:**
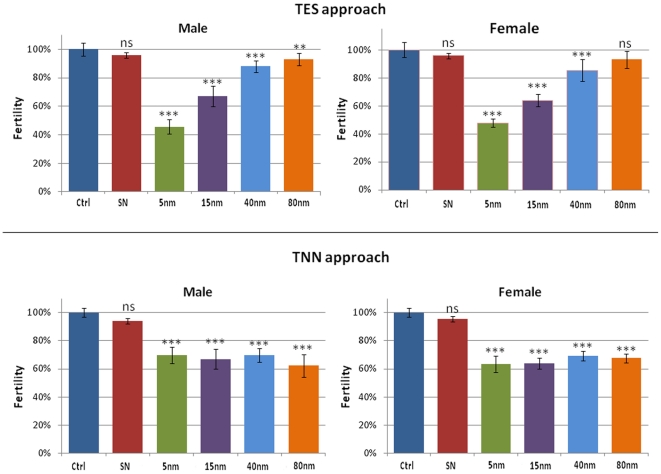
Male (left) and female (right) fertility tests relative to TES (top) and TNN experiments (bottom). Experimental points represent the average from 10 independent experiments and the error bars indicate the standard deviation (ns  =  non significant, i.e. p-value >0.05; **p-value <0.01; ***p-value <0.001).

The toxicity mechanisms induced by AuNPs ingestion were also evaluated by fertility tests in order to assess whether the AuNPs affect the reproductive performance of the flies. Experimental data indicate that AuNPs influence negatively the reproductive performance (Fig. 2) [45]. The NPs effect is similar in both male and female organisms, suggesting a generic and not sex-linked toxicity of AuNPs. Moreover, it is possible to observe that, in this case, AuNPs toxicity seems to be related to their concentration in the food. In fact, in Fig. 2 (top) relative to the TES experiments, a clear decrease of fertility as a function of AuNPs concentration is evident. In particular, the decrease induced by 5 nm AuNPs is very strong (down to ∼46% with respect to the control organisms). On the other hand, the results obtained from flies nurtured with TNN food show a consistent decrease of fertility, nearly constant for all the NPs sizes, for both male and female flies. In line with the lifespan results, we observed that the toxic effects of AuNPs on the reproductive performance of *Drosophila* are directly related to the concentration of AuNPs and not to their size or surface area.

### ROS generation and TUNEL assay

**Figure 3:**
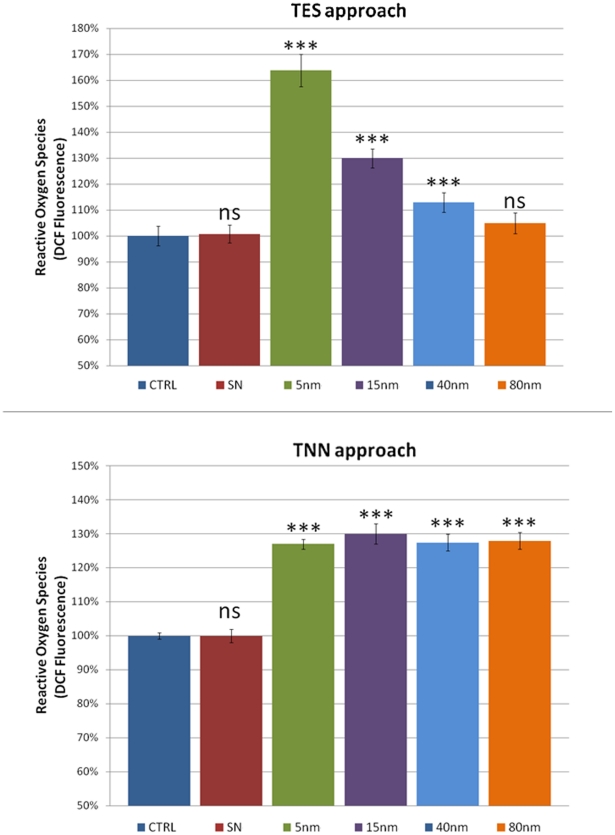
ROS measurements by DCF assay on TES and TNN treatments (top and bottom, respectively). Data are reported as relative fluorescence intensity normalized to the control (ns  =  non significant, i.e. p-value >0.05; ***p-value <0.001). Error bars  =  SD.

We further focused our studies on the generation of ROS in flies treated with AuNPs. In this context, the analysis of ROS level is relevant since some nanoparticles have been shown to induce the formation of ROS *in vitro* [57], [58]. We used the DCFH-DA assay to quantify the ROS levels. Experimental results (Fig. 3) were consistent with the previous observations (see above). In particular, in the TES experiments, we measured high levels of ROS in the 5 nm AuNPs treated flies (c.a. 165% as compared to the control and SN treatment) while in the larger sizes a decrease of the ROS, down to the control level, was observed. Hence, also the trend of the ROS level is primarily governed by the concentration of the NPs. This finding is further confirmed by the TNN experiments in which the ROS level remains constant (∼130% with respect to the control) in all the differently sized AuNPs. Although the exact mechanism of ROS generation by NPs is still unclear at the moment, it has been hypothesized that NPs of different chemical compositions seem to interact with mitochondria, which are redox active organelles, thereby causing interference in the biological antioxidant defense [59], [60]. ROS are important tissue signaling components, and high levels of ROS are generally considered as deleterious to cells [61]. Indeed, above-physiological levels of ROS typically lead to acceleration in ageing, age-related diseases, as well as cell death. They can also constitute a stress signal that activates redox-sensitive signaling pathways. The maintenance of physiological levels of ROS is crucial for normal growth and metabolism [62].

**Figure 4:**
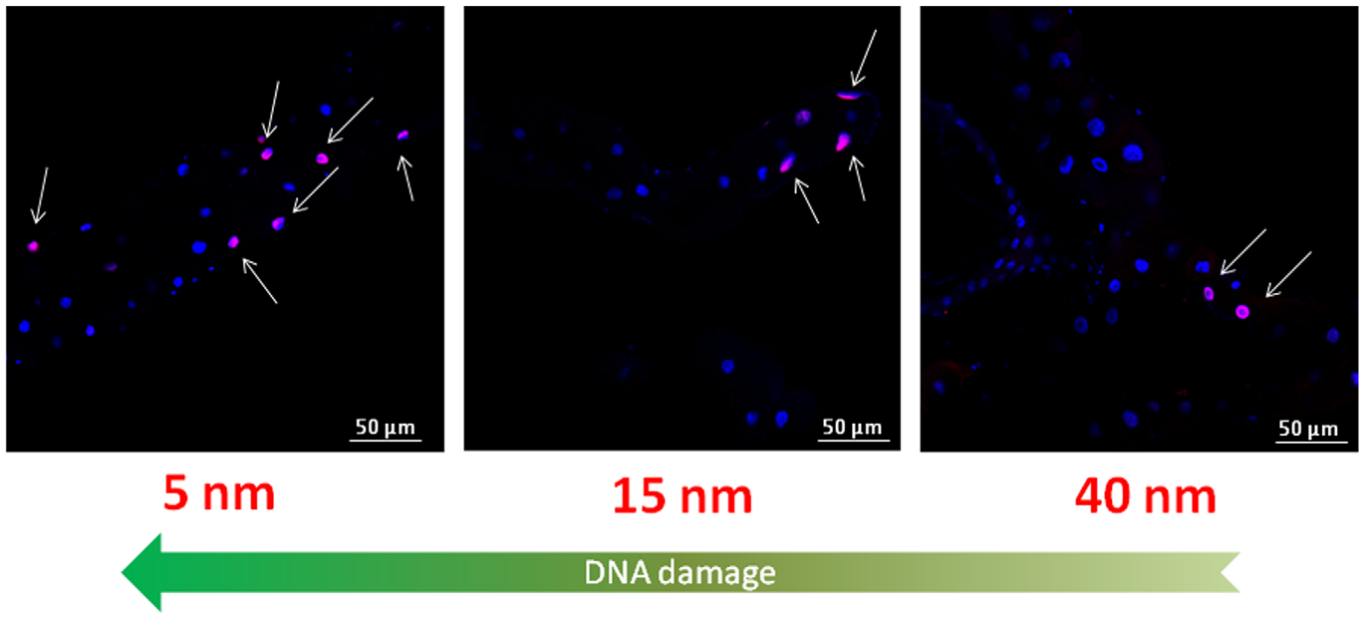
Representative confocal microscopy images of Drosophila midgut in flies obtained from TES treatment. Nuclei are stained with Hoechst 33342 (blue) while cells containing DNA strand nicks are detected by TUNEL assay and fluoresce red (highlighted by the white arrows).

TUNEL assay was also performed to evaluate the possible presence of DNA damage induced by the AuNPs. The results show a strong adverse effect of AuNPs (Fig. 4) [45], highlighting the genotoxic potential of the differently sized AuNPs on the intestinal tissue of *Drosophila*. In particular, in Fig. 4 we observed, for the TES treatment, a significant number of TUNEL positive nuclei for the 5 nm NPs, while DNA fragmentation was found to decrease for bigger NPs (that are less concentrated). For the 80 nm treatment (the lowest concentration), we could not observe detectable DNA damage. A quantitative analysis of TUNEL assay is reported in Figure S3 (results were consistent with previous experiments). However, in the TNN experiments (Figure S3, bottom) we found the occurrence of positive nuclei similar for 5 and 15 nm, while in the case of larger NPs a slight decrease of genotoxic effects was observed. This suggests that, in the specific case of DNA damage in the GI tract, the size of the NPs plays a certain role. This might be ascribed to a more efficient tissue penetration by smaller NPs [63]–[65], with consequent damage to the genetic material. However, since 15 nm NPs typically exhibit cytoplasmic distribution with no detectable penetration in the nuclei, it is likely that the observed DNA fragmentation is the result of indirect interaction of NPs with DNA. In any case, this point deserves further investigations, such as tissue-specific ROS level measurements.

### mRNA expression levels by RT-qPCR

**Figure 5:**
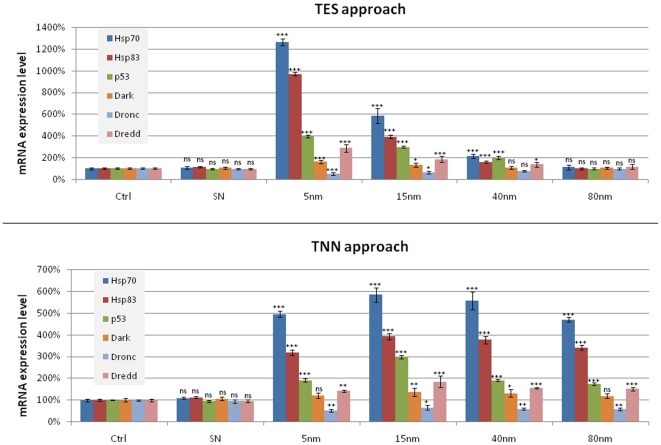
mRNA expression level analyzed by RT-qPCR of *Drosophila* treated with TES (top) and TNN (bottom) approaches. All data relative to RT-qPCR experiments were analyzed by statistical software to evaluate the significant difference with respect to the control (ns  =  non significant, i.e. p-value >0.05; *p-value <0.05; **p-value <0.01 ***p-value <0.001).

To get a deeper insight into the molecular mechanisms underlying the toxic effects of AuNPs, we performed RT-qPCR experiments to analyze the expression profile of some gene involved in the response to stress stimuli (*hsp70* and *hsp83*), DNA damage checkpoints (*p53*) and apoptosis (*Dark, Dronc,* and *Dredd*). Also in this case, the RT-qPCR results relative to the TES and TNN approaches follow the same pattern observed in the previous experiments, supporting the concept of a concentration-dependent toxicity of AuNPs (Fig. 5). In particular, in the TES experiments, the mRNA expression level of *hsp70* and *hsp83* was very high for the 5 nm AuNPs treatment, while the 80 nm treatment was comparable to the control and SN; on the other side, in the TNN approach, their expression level remained similar for all AuNPs sizes. *hsp70* is one of the highly conserved genes and is the first to be induced in *Drosophila* [66], [67] against various physical [68], physiological and chemical stressors [69], [70]; *Drosophila* Hsp83 (homologue of Hsp90 in mammals) works as a chaperone refolding protein system, sometimes in coordination with Hsp70 [71], [72]. A significant induction of both Hsps has been observed in many organisms, upon exposure to heavy metals, demonstrating their role as stress biomarkers [73], [74]. This cellular response was also observed in human population after exposure to various environmental stresses [75]. The results about *hsp70* and *hsp83* expression levels obtained in our experiments indicated the presence of a concentration-dependent general stress due to the AuNPs and clarify the effects observed in lifespan and fertility tests. In fact, the *hsp* genes are known to be strictly associated to the reproductive performance and longevity in *Drosophila* [76], [77].

We also investigated the expression level of *p53* gene, because the p53 pathway is critical to maintain the integrity of the genome in multicellular organisms. The overexpression of *p53* observed in our experiments is in line with the above TUNEL results, indicating the activation of cellular response following the occurrence of significant DNA damage. P53 was found to be over-expressed in response to several types of DNA damage, such as after exposure to genotoxic agents, radiation, ROS formation, or inappropriate oncogene activation [78]–[80]. In particular, in our experiments, the expression of *p53* was significantly increased, especially in the case of 15 nm AuNPs treatment (Fig. 5, bottom). Probably, the AuNPs of 15 nm can induce a secondary effect that has repercussions on the same molecular mechanism, which in turn induces the overexpression of p53. Furthermore, *p53* encodes a transcription factor [81] that activates genes that arrest cell growth and induce apoptosis [82], thereby preventing the propagation of genetically damaged cells. *p53* is the most important tumor suppressor gene known to date: perhaps half of all human neoplasms have mutations in *p53*, and there is a remarkable agreement between oncogenic mutations and the loss of *p53* transcriptional activity [78], [79], [83]. Interestingly, a disturbed level of *p53* has been demonstrated to affect ageing and longevity both in mouse and *Drosophila* [84]–[87], so it might be possible that the increased levels of *p53* detected in our experiments have a role in the NPs-induced decrease of the lifespan of the treated organisms. We analyzed also the expression level of some genes involved in the apoptotic pathway (*Dark, Dredd* and *Dronc*). Dark (*Drosophila* Apaf-1-related killer) is a *Drosophila* CED-4/Apaf-1 homologue; it is an important apoptosis effector in *Drosophila* and raises profound evolutionary considerations concerning the relationship between mitochondrial components and the apoptosis-promoting machinery [88]. Dronc and Dredd represent the initiator caspases in *Drosophila* [89]. Moreover, Dredd (similar to human caspase-8) appears to be mainly involved in the innate immune response pathway [90], whereas Dronc is similar to caspase-9, the apical mammalian caspase involved in stress-mediated apoptosis. Dronc is also required for DNA damage by radiation-induced cell death [91]. In our RT-qPCR experiments (TNN approach) *Dronc* shows a constant downregulation (about 50% with respect to the control), while *Dark* does not exhibit any particular modifications in the expression level, remaining similar to the control for all the AuNPs sizes. On the other hand, *Dredd* shows a constant upregulation for all the AuNPs sizes (Fig. 5, bottom). The observed downregulation of Dronc is likely to be due to the presence of high levels of Hsps that are demonstrated to inhibit apoptosome formation and/or recruitment of caspase-9 to the complex by binding to cytochrome *c* or Apaf-1 [92]. However, the upregulation of Dredd confirms the presence of apoptosis event in *Drosophila* and opens new dramatic questions about the activation of the innate immune response pathway due to the stress induced by the AuNPs.

### Conclusions

In this work, we have demonstrated the *in vivo* toxicity of citrate capped AuNPs of different sizes (5, 15, 40, and 80 nm), upon the physiological administration route of ingestion, on the model organism *Drosophila melanogaster*. In particular, by using two different approaches (TES and TNN), we assessed that, in the 5–80 nm size range, the concentration of the AuNPs plays a primary role in determining the toxic effects, while the size (surface) of the AuNPs does not seem to be a key parameter. Lifespan and fertility tests showed a clear concentration dependent reduction of *Drosophila* viability and reproductive performance, indicating a general, not sex-linked, stress in the whole organism. Moreover, ROS level measurements indicated the presence of adverse effects also at cellular level, with possible consequences in ageing and age-related diseases, DNA damage and cell death. The TUNEL assay revealed a significant AuNPs induced DNA damage, highlighting the genotoxic effects induced by the differently sized AuNPs on the intestinal tissue of *Drosophila*. Finally, the RT-qPCR experiments validated the concentration-dependent toxicity of the AuNPs, evidencing the presence of generalized stress (*hsp70* and *hsp83*), DNA damage (*p53*), and apoptotic events (*Dronc*). On the other side, the observed down-regulation of *Dredd* opens new questions about the possible activation of immune response in *Drosophila melanogaster.* Overall, our results indicate a significant concentration-dependent, size-independent *in vivo* toxicity of citrate capped AuNPs in *Drosophila*, corroborating the emerging picture of remarkable toxicity of naked AuNPs [30], as opposed to protein/polymer coated or nanoscale surface engineered AuNPs [93]. In this respect, although the molecular mechanisms underlying AuNPs toxicity are not well clarified so far, specific protein/polymer coatings surrounding the nanoparticles are likely to play a protective role, avoiding direct NP/biomolecule interactions and/or intracellular ions release, which may promote the alteration of downstream processes, including ROS overproduction.

## Supporting Information

Figure S1 (A–D) Representative TEM images of 5,15, 40, and 80 nm citrate-capped AuNPs; in the table are listed the NPs features obtained from different characterization techniques, namely size distribution analysis from more than 100 NPs imaged by TEM in random fields, hydrodynamic diameter and polydispersion index (PdI) obtained from DLS measurements, and Z-potential analysis. The observed Z-potential values are in line with the expected negatively charged surface area of the NPs, due to citrate capping.
(TIF)

Figure S2 Representative TEM images of (A) 15 nm and (B) 80 nm AuNPs mixed with the *Drosophila* food.
(TIF)

Figure S3 Quantitative analysis of TUNEL positive nuclei relative to TES (top) and TNN experiment (bottom). Experimental points represent the average of data from 20 microscopic fields of 3 independent experiments and the error bars indicate the standard deviation (ns  =  non significant; *p-value <0.05; **p-value <0.01)
(TIF)

Table S1 Surface area, molar concentration, number of nanoparticles, mass of AuNPs in food and mass of AuNPs ingested from *Drosophila* per day relative to each size of AuNPs for TES (up) and TNN (bottom) approach.
(TIF)

Table S2 List of primers used in RT-qPCR experiments. All primers were designed using on-line NCBI Primer-BLAST software.
(TIF)

Table S3 Statistical analyses of the TES and TNN lifespan curves (top and bottom, respectively). TES statistical analyses reveal a significant difference between all the treatments compared to the control (CTRL). The comparison between CTRL and SN reveals a non significant difference (p-value >0.05). TNN statistical analyses reveal an effective difference between all the treatments compared to the control (CTRL). The comparison between the treatments reveals a non significant difference (p-values >0.05)
(TIF)
