# Supplementary figures and images for: Concentration-Dependent, Size-Independent Toxicity of Citrate Capped AuNPs in Drosophila melanogaster
Source: PLoS One. 2012 Jan 4;7(1):e29980. doi: 10.1371/journal.pone.0029980 (PMC3251612; doi:10.1371/journal.pone.0029980)

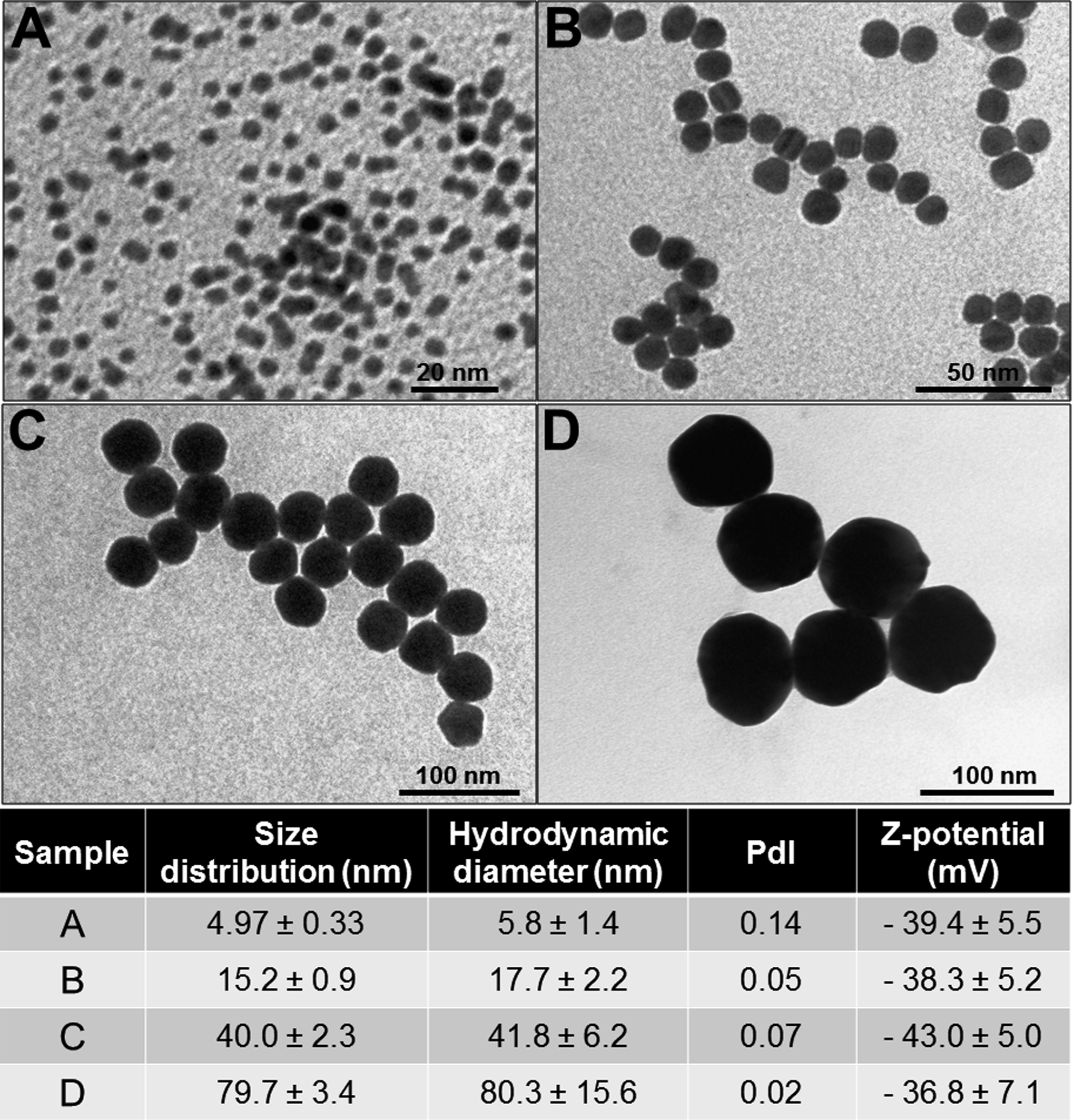

Supplement: Figure S1 — (A–D) Representative TEM images of 5,15, 40, and 80 nm citrate-capped AuNPs; in the table are listed the NPs features obtained from different characterization techniques, namely size distribution analysis from more than 100 NPs imaged by TEM in random fields, hydrodynamic diameter and polydispersion index (PdI) obtained from DLS measurements, and Z-potential analysis. The observed Z-potential values are in line with the expected negatively charged surface area of the NPs, due to citrate capping. (TIF) [file pone.0029980.s001.tif]

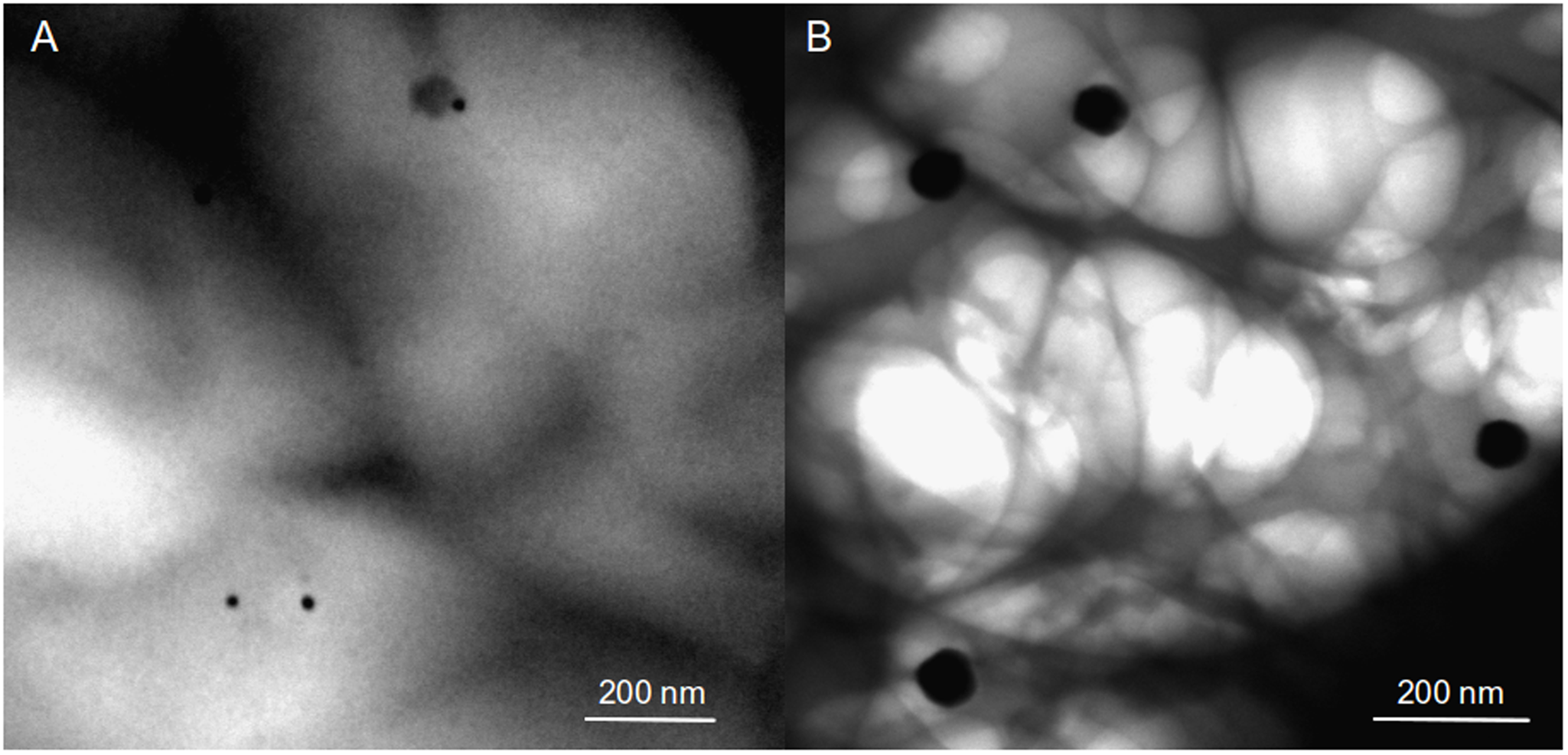

Supplement: Figure S2 — Representative TEM images of (A) 15 nm and (B) 80 nm AuNPs mixed with the Drosophila food. (TIF) [file pone.0029980.s002.tif]

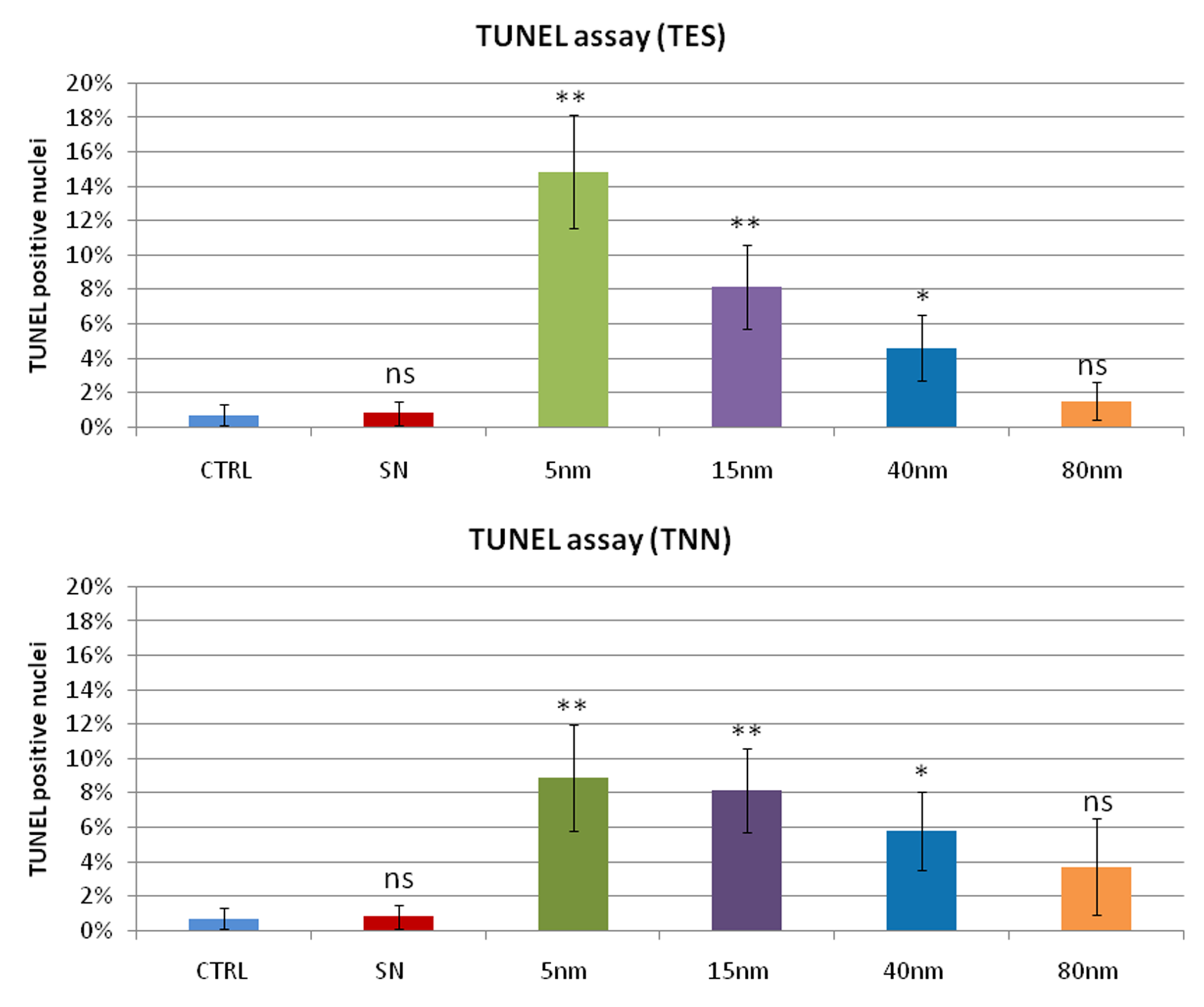

Supplement: Figure S3 — Quantitative analysis of TUNEL positive nuclei relative to TES (top) and TNN experiment (bottom). Experimental points represent the average of data from 20 microscopic fields of 3 independent experiments and the error bars indicate the standard deviation (ns = non significant; *p-value <0.05; **p-value <0.01) (TIF) [file pone.0029980.s003.tif]

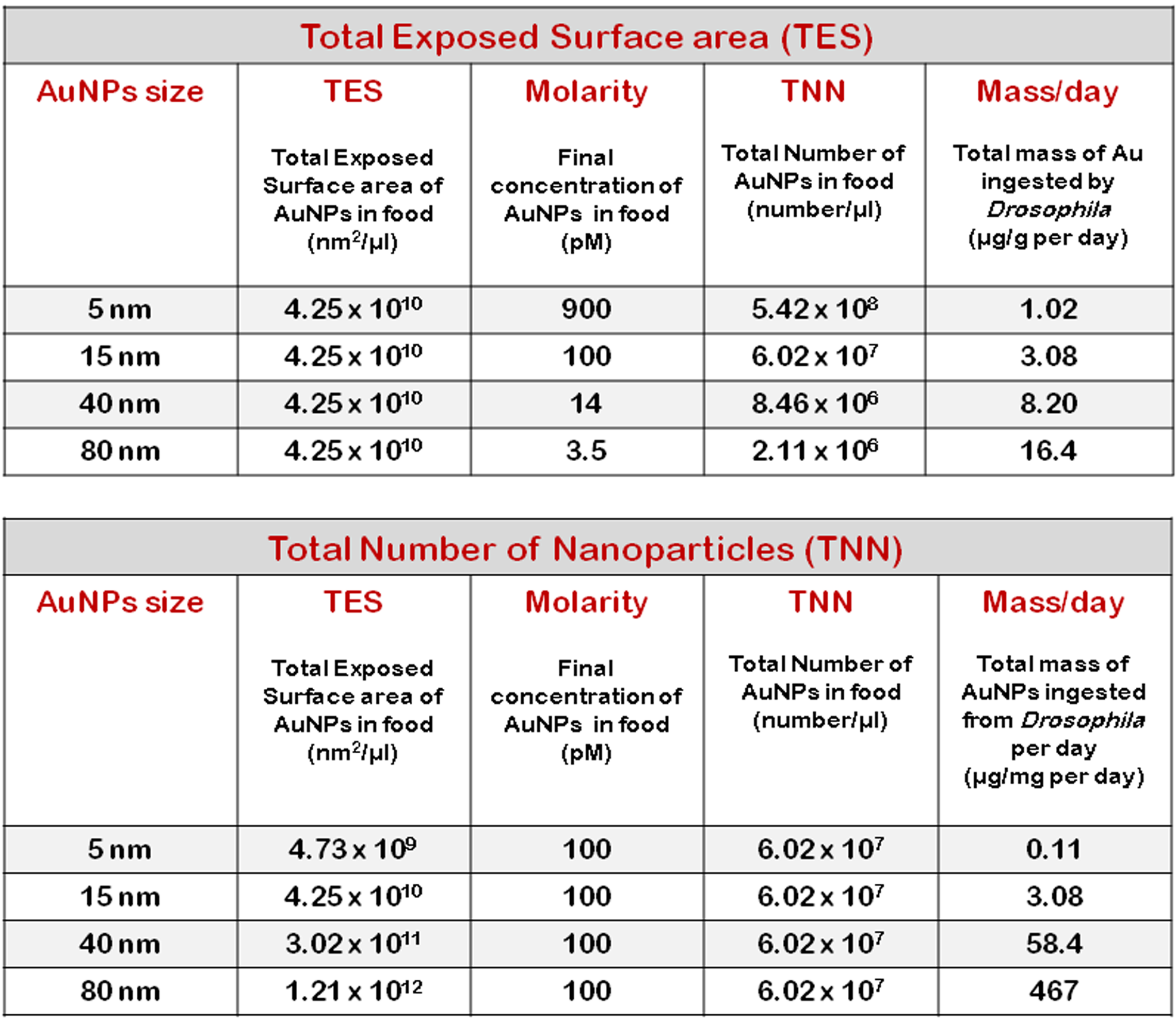

Supplement: Table S1 — Surface area, molar concentration, number of nanoparticles, mass of AuNPs in food and mass of AuNPs ingested from Drosophila per day relative to each size of AuNPs for TES (up) and TNN (bottom) approach. (TIF) [file pone.0029980.s004.tif]

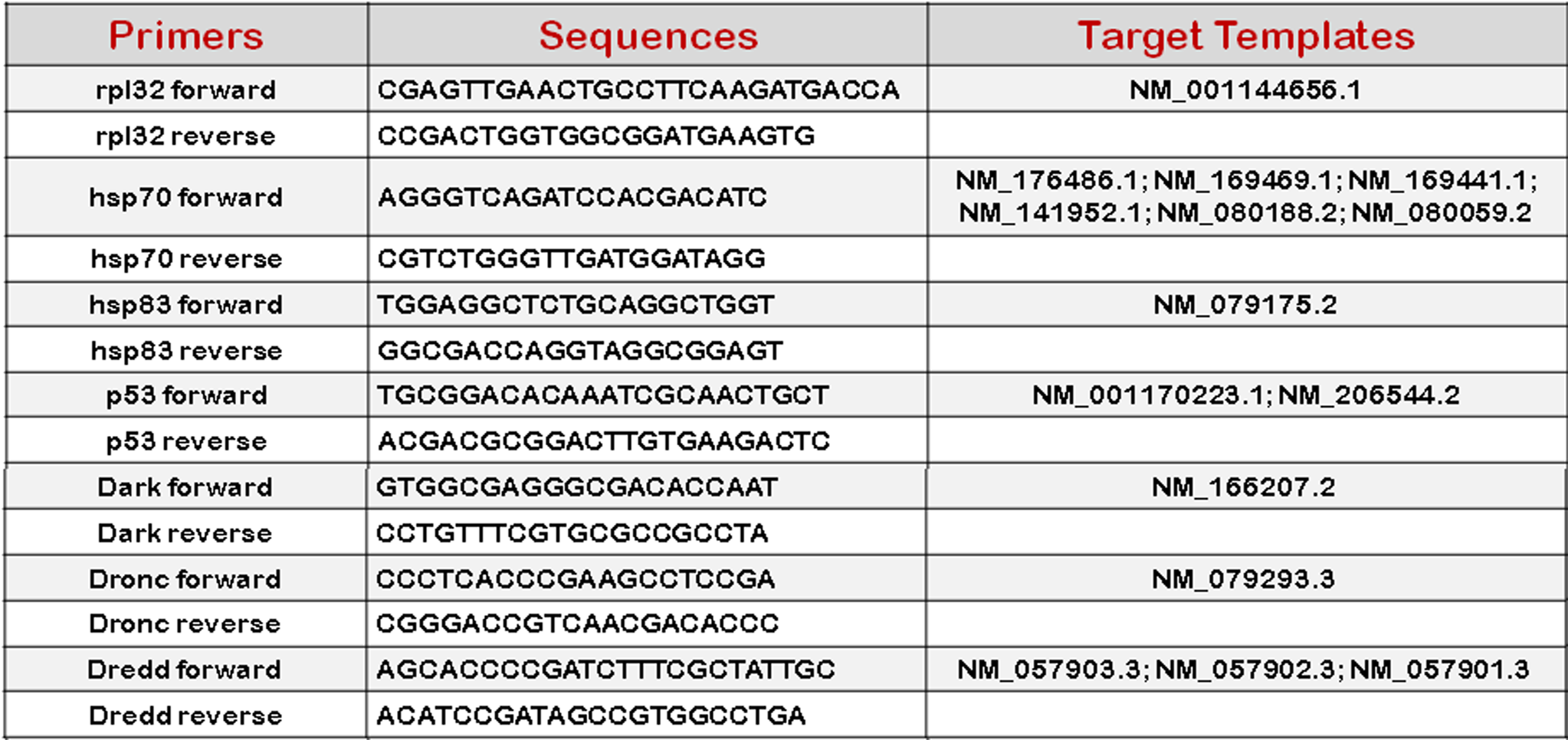

Supplement: Table S2 — List of primers used in RT-qPCR experiments. All primers were designed using on-line NCBI Primer-BLAST software. (TIF) [file pone.0029980.s005.tif]

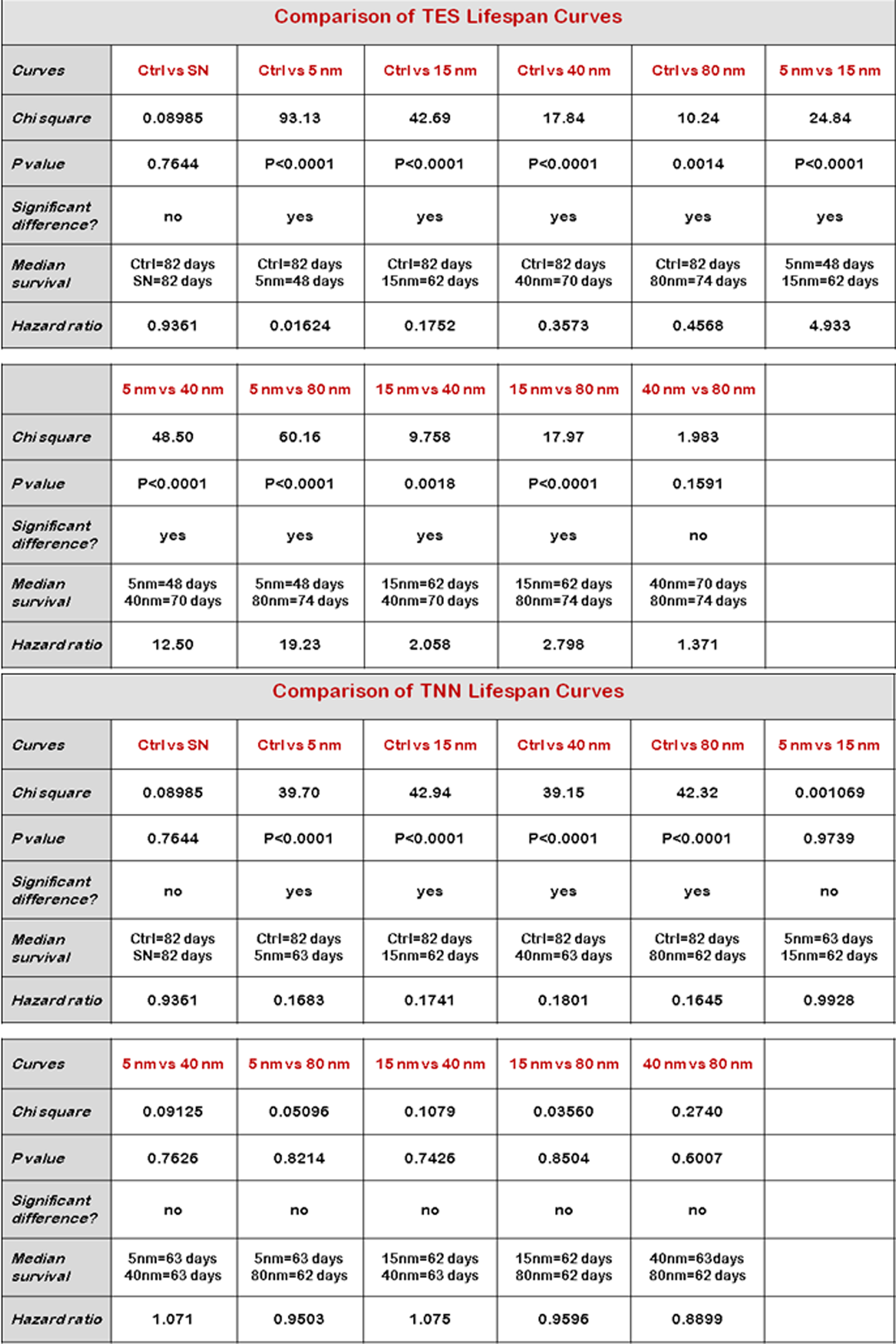

Supplement: Table S3 — Statistical analyses of the TES and TNN lifespan curves (top and bottom, respectively). TES statistical analyses reveal a significant difference between all the treatments compared to the control (CTRL). The comparison between CTRL and SN reveals a non significant difference (p-value >0.05). TNN statistical analyses reveal an effective difference between all the treatments compared to the control (CTRL). The comparison between the treatments reveals a non significant difference (p-values >0.05) (TIF) [file pone.0029980.s006.tif]
